# Smoking-associated increase in mucins 1 and 4 in human airways

**DOI:** 10.1186/s12931-020-01498-7

**Published:** 2020-09-18

**Authors:** Heta Merikallio, Riitta Kaarteenaho, Sara Lindén, Médea Padra, Reza Karimi, Chuan-Xing Li, Elisa Lappi-Blanco, Åsa M. Wheelock, Magnus C. Sköld

**Affiliations:** 1 Respiratory Medicine Unit, Department of Medicine Solna and Center for Molecular Medicine, Karolinska Institutet, Stockholm, Sweden; 2 Research Unit of Internal Medicine, University of Oulu, Oulu, Finland; 3 Medical Research Center Oulu, Oulu University Hospital, Oulu, Finland; 4 Department of Biomedical Chemistry and Cell Biology, Institute of Biomedicine, Sahlgrenska Academy, University of Gothenburg, Gothenburg, Sweden; 5 Department of Respiratory Medicine and Allergy, Karolinska University Hospital Solna, Stockholm, Sweden; 6 Department of Pathology, Center for Cancer Research and Translational Medicine, Medical Research Center Oulu, Oulu University Hospital and University of Oulu, Oulu, Finland

**Keywords:** Bronchus, Epithelium, Mucin, Smoking, COPD, Chronic Bronchitis

## Abstract

**Rationale:**

Smoking-related chronic obstructive pulmonary disease (COPD) is associated with dysregulated production of mucus. Mucins (MUC) are important both for mucus secretion and epithelial defense. We have examined the distribution of MUC1 and MUC4 in the airway epithelial cells of never-smokers and smokers with and without COPD.

**Methods:**

Mucosal biopsies and bronchial wash samples were obtained by bronchoscopy from age- and sex-matched COPD-patients (*n* = 38; GOLD I-II/A-B), healthy never-smokers (*n* = 40) and current smokers with normal lung function (*n* = 40) from the Karolinska COSMIC cohort (NCT02627872). Cell-specific expressions of MUC1, MUC4 and regulating factors, i.e., epithelial growth factor receptor (EGFR) 1 and 2, were analyzed by immunohistochemistry. Soluble MUC1 was measured by quantitative immunodetection on slot blot.

**Results:**

The levels of cell-bound MUC1 expression in basal cells and in soluble MUC1 in bronchial wash were increased in smokers, regardless of airway obstruction. Patients with chronic bronchitis had higher MUC1 expression. The expression of MUC4 in cells with goblet cell phenotype was increased in smokers. The expression of EGFR2, but not that of EGFR1, was higher in never-smokers than in smokers.

**Conclusions:**

Smoking history and the presence of chronic bronchitis, regardless of airway obstruction, affect both cellular and soluble MUC1 in human airways. Therefore, MUC1 may be a novel marker for smoking- associated airway disease.

## Introduction

The mucus layer on the top of the airway epithelium forms the first line of defense against pathogens, toxins and foreign particles [1]. Mucus binds and clears the pathogens through mucociliary clearance. However, abnormal mucus production and clearance can contribute to respiratory diseases such as chronic obstructive pulmonary disease (COPD) [2–4].

Mucin (MUC) macromolecules are believed to participate in the mucosal defense system by protecting the airway epithelium [5, 6]. Moreover, increased numbers of mucus producing cells i.e. hyperplasia of goblet cells, in the airway epithelium have been shown to associate with increased production of MUC [5]. MUCs are high molecular weight glycosylated proteins and are key components of most gel-like secretions [7]. Airway MUCs are major constituents of the secreted layer that comprise lung mucus in healthy airways and are a part of the mucociliary defense system that protects the lungs [8]. Many mucins, e.g. MUC1, − 2, − 3, − 4, − 5 AC, −5B, − 6, − 7, − 8, − 13, and − 19 have been detected in the lung. Among these, MUC1, MUC4 and MUC16 are transmembrane mucins with a large external polypeptide core protein [9–11]. Human MUC1 is expressed in two different forms, as a transmembrane protein complex and as a secreted isoform [12]. Transmembrane MUC1 can also be cleaved from the epithelial surface by sheddases [13]. The initiation of mucin secretion involves a secretory cascade that results in a rapid release of MUCs both from the airway epithelial cells and secretory cells in the submucosal glands. MUC production is regulated by receptors for certain growth factors, e.g. epidermal growth factor receptor (EGFR) and 2 (EGFR2), also known as HER2 or ErB2 [14–16].

MUC1 was originally thought to be expressed in most secretory human epithelial cells [17]. In histopathology, MUC1 has been commonly referred to as epithelial membrane antigen (EMA), or episialin, while its secreted form is also known as the Krebs von den Lungen antigen (KL-6) [17]. MUC1 has been shown to affect tumor progression in cancers in many organs e.g. a high expression of MUC1 has also been detected in lung cancer [9, 18]. Furthermore, MUC1 can act as a releasable decoy and limit the number of bacteria that access the epithelial cells [13]. Membrane-bound MUC4 also seems to be expressed in the lung [18]. The level of MUC4 is elevated in lung cancer and it has been speculated to play various diverse roles in tumorigenesis [19]. The β-subunit of MUC4 is able to interact with EGFR2 [20].

We hypothesized that the localization and expression of MUC1 and MUC4 in human airways would differ between smokers with or without COPD, and that a difference would also be evident in the levels of soluble MUC1. To test these hypotheses, we investigated bronchial mucosal biopsies and bronchial secretions from never-smokers, smokers with normal lung functions and patients with COPD from the Karolinska COSMIC cohort. For mechanistic purposes, we also evaluated the expression of EGFR1 and EGFR2.

## Material and methods

### Study subjects and patients

The investigations were performed on specimens from the Karolinska COSMIC cohort (Clinical & Systems Medicine Investigations of Smoking-related Chronic Obstructive Pulmonary Disease) (www.ClinicalTrials.gov/ct2/show/study/NCT02627872). The cohort has been described in detail previously [21–30]. The demographics of the included subjects are summarized in Table 1. Briefly, we investigated healthy never-smokers (Never-smokers, *n* = 40), current smokers with normal lung function (Smokers, *n* = 40), and patients with mild-to-moderate COPD (*n* = 38) of GOLD stage I – II/A-B (FEV_1_/FVC < 0.7 and FEV_1_ 50–100% of predicted) including both current smokers and ex-smokers. The groups were matched in terms of age (45–65 years), gender, as well as smoking history (> 10 pack-years) and current smoking habits (smoking (> 10 cigarettes/day past 6 months)) with the relevant groups. The smoking status of the latter was verified by assay of exhaled carbon monoxide [31]. Chronic bronchitis was diagnosed in 19 patients [32].

**Table 1:** Clinical data of the study subjects

	Never-smokers	Current Smokers	Current Smokers with COPD	Ex-smokers with COPD
**n**	40	40	27	11
**Male|Female**	20|20	20|20	15|12	5|6
**Age**	57 (6.99)	54 (6.10)	59 (5.05)	60 (5.55)
**Pack years**	0 (0)	35.2 (12.4)	42.1 (10.2)	29.4 (9.1)
**Cigarettes/day past 6 month**	0	17.8 (6.61)	16.5 (6.44)	0
**FEV1 (% of predicted)**	118 (12.8)	109 (11.9)	78.7 (10.5)	78.6 (14.6)
**FEV1/FVC**	0.81 (0.54)	0.78 (0.47)	0.61 (0.60)	0.60 (0.78)
**FEV1/VC**	0.80 (0.74)	0.76 (0.63)	0.60 (0.90)	0.56 (0.11)
**DLCO (% of predicted)**	92.5 (11.3)	79 (12.2)	66.4 (12.5)	69.5 (16.2)
**TLC (L)**	6.56 (1.23)	6.52 (1.28)	6.66 (1.19)	6.60 (1.04)
**Chronic Bronchitis (n)**	0	10	7	2

Data is shown as mean and standard deviation. *FEV1* Forced expiratory volume in 1 s, *FVC* Forced vital capacity, *VC* Vital capacity, *DLCO* Diffusing Capacity for Carbon Monoxide, *TLC* Total Lung Capacity

### Bronchoscopy, biopsy retrieval and bronchial wash samples

Bronchoscopy was performed as previously described [33–35]. Biopsy specimens were taken by pulmonary biopsy forceps with smooth-edged jaws (Radial Edge® Biopsy Forceps, Boston Scientific, Boston, MA). Four to six bronchial biopsies were retrieved from each study patient, and they were collected from lobar or segmental carinae of the upper lobes or the apical segment of the lower lobes. All biopsies were immediately formalin-fixed and embedded in paraffin. The tissue samples were stained with haematoxylin-eosin (HE) and a preceding quality evaluation was performed, with the representativeness all biopsies being evaluated. Two representative tissue blocks from each case were selected for immunohistochemical studies for MUC1, MUC4, EGFR1 and EGFR2. Staining was performed in consecutive sections. p63 (for basal cells) and Alcian-Blue periodic acid-Schiff (AB-PAS) (for goblet cells) staining were performed for phenotyping of epithelial cells.

Bronchial wash samples were obtained by instilling 10 mL of sterile phosphate-buffered saline (PBS) at 37 °C into a segmental bronchus in the right upper lobe, after which the fluid was gently suctioned back. Samples were frozen without filtration or centrifugation, and stored at − 80 °C until use.

### Immunohistochemical staining and quantification of the expression for MUC1, MUC4, EGFR1 and EGFR2

Four μm thick sections were cut from the paraffin embedded tissue blocks, deparaffinized with xylene and rehydrated in a descending ethanol series. The primary antibodies used in the immunostaining were tested for formalin fixed paraffin embedded tissues. The antibodies used are summarized in Table 2. All antibodies were stained with DAKO REAL EnVision-kit from Dako (Dako, Glostrup Denmark). Before application of the primary antibodies for MUC1 and EGFR1, the sections were heated in a microwave oven in 10 mM citrate buffer, pH 6.0, for 10 min. MUC4 and EGFR2 epitopes were retrieved by heating with Tris-EDTA, pH 9.0 for 10 min. After overnight incubation at + 4 °C with the primary antibody (Table 2), a biotinylated secondary HRP Rabbit/mouse -antibody (Dako, Envision) was used. In all the immunostainings the colour was developed with diaminobenzidine (DAB), subsequently the sections were lightly counterstained with haematoxylin. To identify the phenotype of the airway cells, the consecutive sections were also stained with a commercially available antibody against p63 (basal cells, Novocastra, NCL-p63) and a histological Alcian Blue-Periodic acid-Schiff stain (AB-PAS, goblet cells) (Supplemental Fig. 1). Negative control stainings were carried out by substituting non-immune rabbit or mouse primary antibody isotype control (Zymed Laboratories Inc. South San Francisco, CA) and PBS for the primary antibodies.

**Table 2:** Antibodies used in immunohistochemical stainings

Antibody	Producer| Clone	Kit	Antigen retrieval	Dilution
MUC1	Novocastra. cloneMa695	Envision	Citrate pH 6	1/ 100
MUC4	Invitrogen. clone IG8	Envision	Tris- EDTA pH 9	1/ 100
EGFR1	Novocastra. NCL-L-EGFR_384	Envision	Citrate pH 6	1/ 100
EGFR2	Novocastra. c-erb-2 oncoprotein	Envision	Tris- EDTA pH 9	1/ 500

In the evaluation of immunohistochemical samples, cytosolic positivity was considered significant; in addition EGFR was also nuclear positive but this was not recorded. The intensity of immunostaining was assessed as 0 (negative), 1 (faintly positive), 2 (positive), 3 (strongly positive) and 4 (very strongly positive), and the extent of the positive staining was estimated from 0 to 100% in each cell type present in the airways i.e. basal cell, goblet cell and respiratory cell (ciliated and non-ciliated). The score for each antibody was calculated by multiplying the total intensity with the extent, resulting in a total score with a range between 0 and 400 [18, 36]. The evaluation was performed blinded to the clinical information of the study subjects by an experienced researcher (HM). Sixty percent of the samples were also evaluated by a pulmonary pathologist (RiK). According to Cohen’s kappa (Ƙ) coefficient, the intra-class correlation between the two assessments was 0.72 and categorised as substantial [37].

### Quantification of soluble MUC1

Complete protease inhibitor cocktail (Sigma p8340) was added to bronchial wash samples (10 μL to 1 mL of sample) during thawing. Samples were diluted 1/100 in reduction buffer (6 M GuHCl, 5 mM EDTA, 0.1 M Tris/HCl, pH 8.0). An aliquot of 100 μL of each sample was loaded onto a PVDF-FL membrane (Millipore, Bedford, MA, USA) using a Minifold–II Slot Blot apparatus (Schleicher & Schuell Bioscience, Germany). In addition, nine serial dilutions of the MUC1 standard (recombinant MUC1 produced in cell culture) were also loaded. A vacuum was applied to attach the MUCs to the membrane. The membranes were then dried for 1 h, pre-wetted briefly in 100% methanol, rinsed with ultrapure water and incubated in phosphate buffered saline (PBS, 0.14 M NaCl, 0.0027 M KCl, 0.010 M PO_4_^3^) for 10 min. Unspecific binding was blocked by incubating in Odyssey blocking buffer (LI-COR Biosciences, NE, USA) for 1 h at 22 °C. Membranes were then incubated with anti-MUC1 monoclonal antibody (BC-2) diluted 1:1000 in Odyssey blocking buffer containing 0.1% Tween 20 overnight at 4 °C with gentle shaking. The membranes were washed four times for 5 min each at 22 °C in PBS containing 0.1% Tween 20 (PBS-T). Thereafter, the membranes were incubated with goat anti-mouse IR dye 800 secondary antibody (LI-COR, Biosciences) diluted 1:10000 in blocking buffer containing 0.1% Tween-20 and 0.01% SDS for 30 min in the dark at 22 °C. The membranes were washed (4 × 5 min) in PBS-T, and blots imaged with the Odyssey infrared imaging system (LI-COR, Biosciences) and quantified with ImageJ software.

### Statistical methods

Statistical analyses were made with IBM SPSS statistics 24 (IBM, Amonk, NY) and with the R-package (version 3.3.3). The Kruskall-Wallis-test was used to compare the immunohistological expression of individual factors. Pearson’s product moment correlation coefficient was applied to test for associations between factors and lung functions. *P*-values less than 0.05 were considered statistically significant. All comparisons between groups were performed with and without stratification by gender, current smoking-status, chronic bronchitis diagnosis, and COPD diagnosis. False Discovery Rate (FDR) method was used for corrections of the multiple testing. FDR less than 0.2 was considered statistically significant (Supplemental Tables 1, 2 and 3).

## Results

### Immunohistochemical findings in airways

**Fig. 1:**
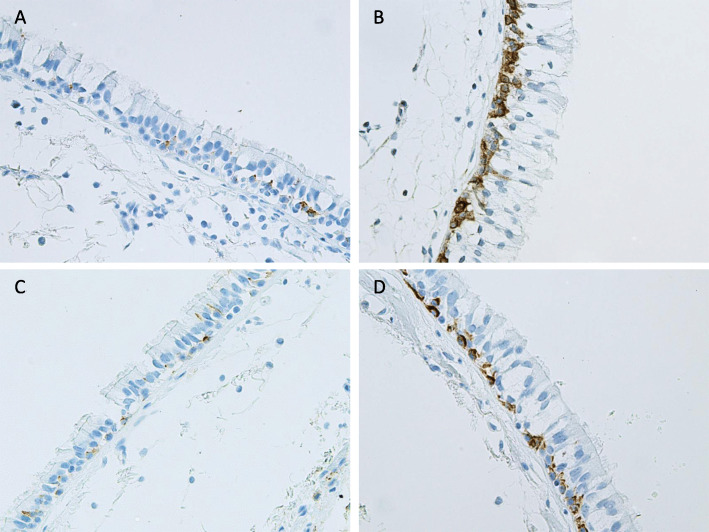
Representative images of the immunohistochemical stainings for cell-bound MUC1 in bronchial biopsy samples. Intensity of the expression was designated as negative, faint, moderate, strong or very strong, and the extent of the positive staining was estimated from 0 to 100% in each cell type present in the airways. Expression of MUC1 in cells suggesting basal cell phenotype in the large airways of a Never-smoker with normal lung functions (**a**), a Smoker with normal lung function (**b**), MUC1 expression in basal cells of an ex-smoker with COPD (**c**) and a Smoker with COPD (**d**)

**Fig. 2:**
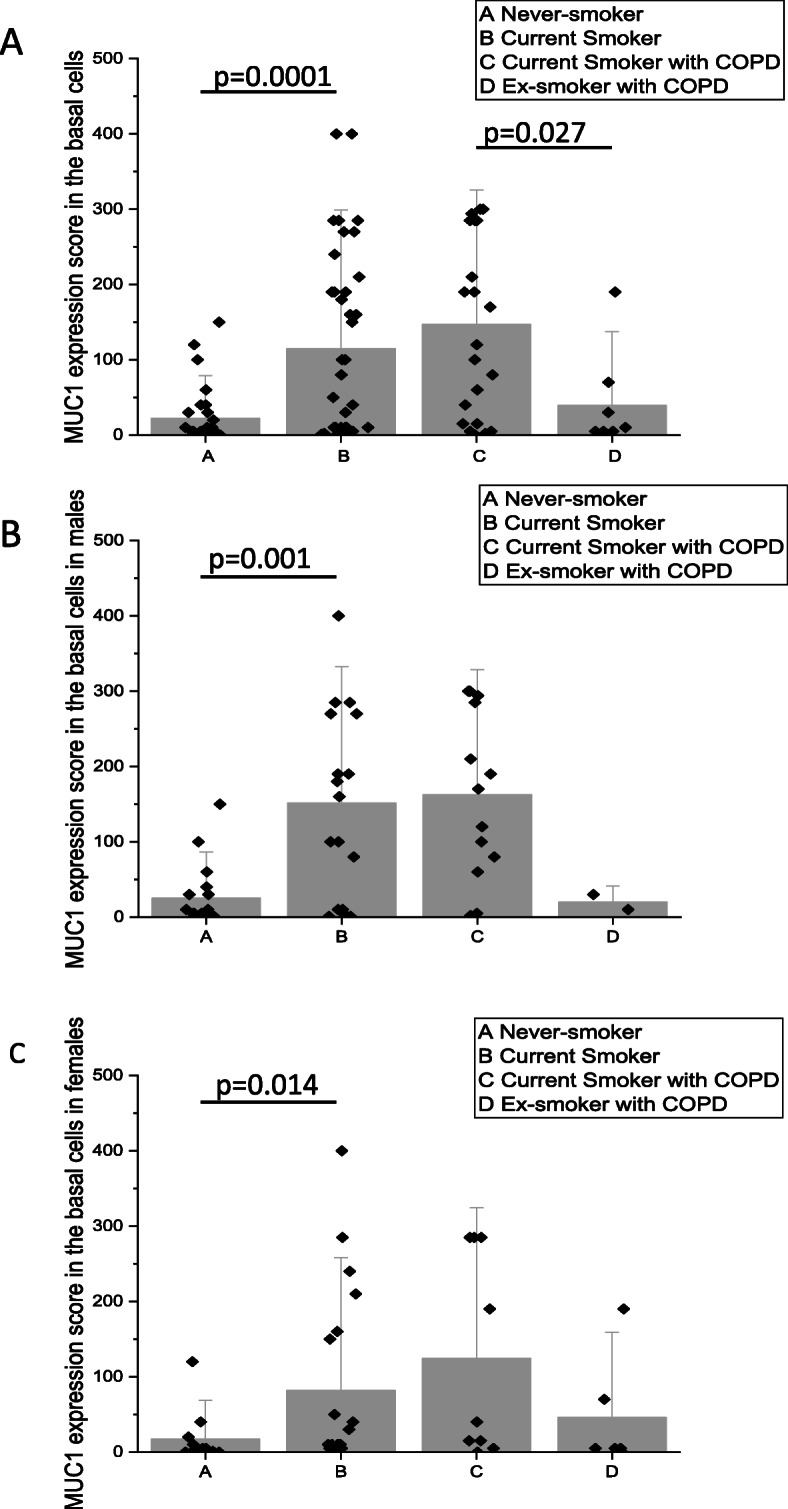
Immunohistochemical scores for cell-bound MUC1 were calculated based on the intensity and extent of the staining. Scores for immunohistochemical expression of MUC1 in basal cells of large airways from smokers (including COPD) and Never-smokers and from ex-smokers with COPD. MUC1 scores in all subjects (**a**), and scores separately in males (**b**) and females (**c**). Results are shown as mean bars with standard error of mean

Cell-bound MUC1 expression, as assessed by immunohistochemistry, was primarily localized to the cytosol of the cells of similar location than those of p63 positive cells. This finding suggest that the basal cells were positive for MUC1. There was, an absence of expression in other types of airway epithelial cells (Fig. 1). Both the intensity and the extent of the immunoreactivity varied between samples (Fig. 1). MUC1 was also positive in the sub-epithelial glands. MUC1 expression was more intense in the Smoker than in the Never-smoker group (*p* = 0.0001) (Fig. 2a), which was also seen following stratification according to gender (males: *p* = 0.0001 (Fig. 2b); females: *p* = 0.014 (Fig. 2c). There were no significant differences in the MUC1 expression between Smokers with COPD and those not suffering from COPD. Smokers with COPD exhibited higher levels of MUC1 expression than ex-smokers with COPD (*p* = 0.027) (Fig. 2a), insignificant difference was detected in males and females (Fig. 2b and c, respectively).

**Fig. 3:**
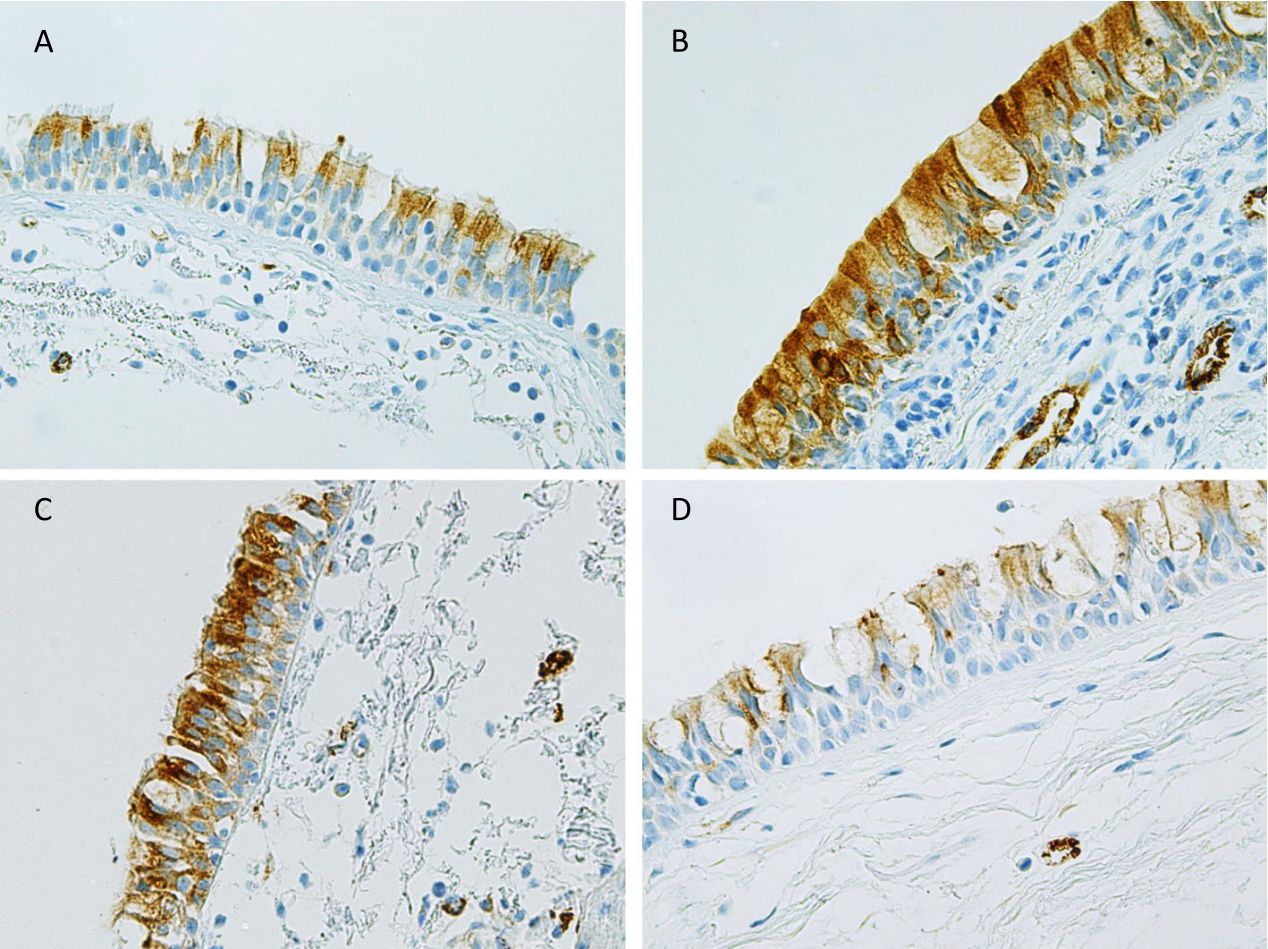
Representative images of the immunohistochemical stainings for MUC4 in bronchial biopsy samples. MUC4 was positive mainly in cells suggesting basal cell and ciliated cell phenotypes, in some cases also in cells with goblet cell phenotype. MUC4 expression in ciliated and basal cells in a Never-smoker (**a**), a positive expression for MUC4 in the main epithelial cell types in a Smoker (**b**), an ex-smoker with COPD (**c**) and a smoker with COPD (**d**)

**Fig. 4:**
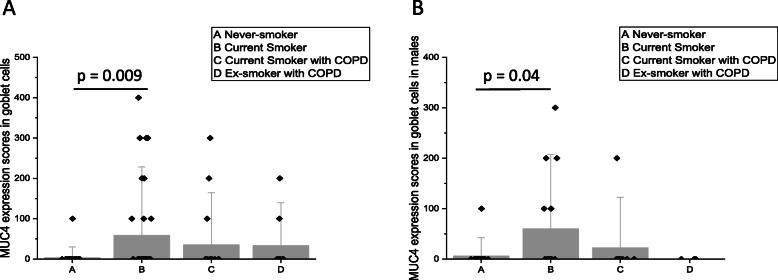
Significant results of the scores on the immunohistochemical expression of MUC4 in goblet cells of large airways. MUC4 scores in the goblet cells of all subjects (**a**) and in males (**b**). Results are shown as median bars with standard error of median

MUC4 was expressed mainly in ciliated cells and basal cells. In 29 of 118 patients, cells suggesting goblet cell phenotype also stained positively for MUC4 (Fig. 3b). MUC4 was also positive in endothelial cells of the blood vessels. The expression level was higher in goblet and basal cell phenotypes in Smokers as compared to Never-smokers (*p* = 0.009 and *p* = 0.047, respectively), where the levels below the detection limit in all except one of the Never-Smokers (Fig. 4). In male Smokers, a higher level of MUC4 expression was observed in cells with goblet cell phenotype in comparison to the Never-smokers (*p* = 0.043) (Fig. 4b). When analyzing the entire cohort, MUC4 expression correlated positively with MUC1 expression in cell with basal cell phenotype (*R* = 0.34, *p* = 0.03), with no pronounced gender difference (males: *R* = 0.36, *p* = 0.01; females: *R* = 0.34, *p* = 0.03). In order to evaluate if there were any morphological differences between MUC1 or MUC4 expressing and non-expressing cells, we re-evaluated 20 biopsies with MUC1 staining and 20 biopsies with MUC4 staining. Half of the re-evaluated samples were scored as low expression (score < 200) while half of them were scored a high expression (score ≥ 200) of MUC1 and MUC4. No morphological differences were observed between samples with high or low expression of the mucins.

**Fig. 5:**
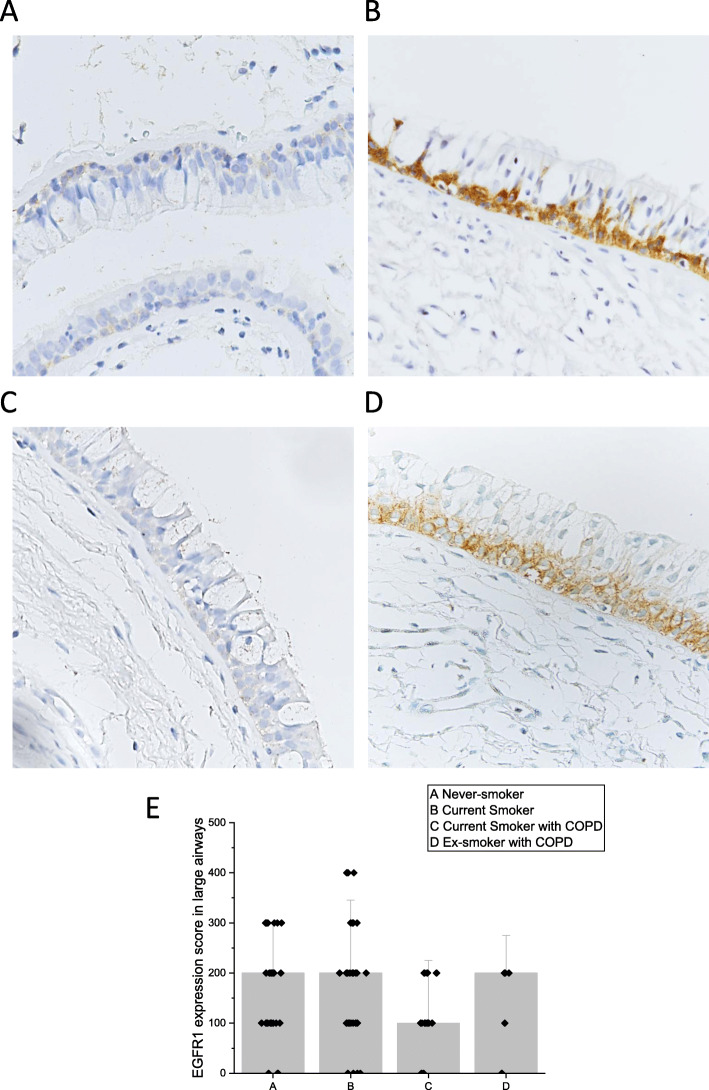
Representative images of the immunohistochemical stainings for EGFR1. Expression of EGFR in cells suggesting basal cell phenotype in the large airways of a Never-smoker with normal lung functions (**a**), a Smoker with normal lung function (**b**), MUC1 expression in basal cells of an ex-smoker with COPD (**c**) and a Smoker with COPD (**d**). Expression scores of EGFR1 in large airways (**e**)

The cytosol of cells suggesting the basal cell phenotype stained positively for EGFR1 (Fig. 5a-d) while other airway epithelial cell types were negative. There were no differences in the expression of EGFR1 between the studied groups (Fig. 5e).

**Fig. 6:**
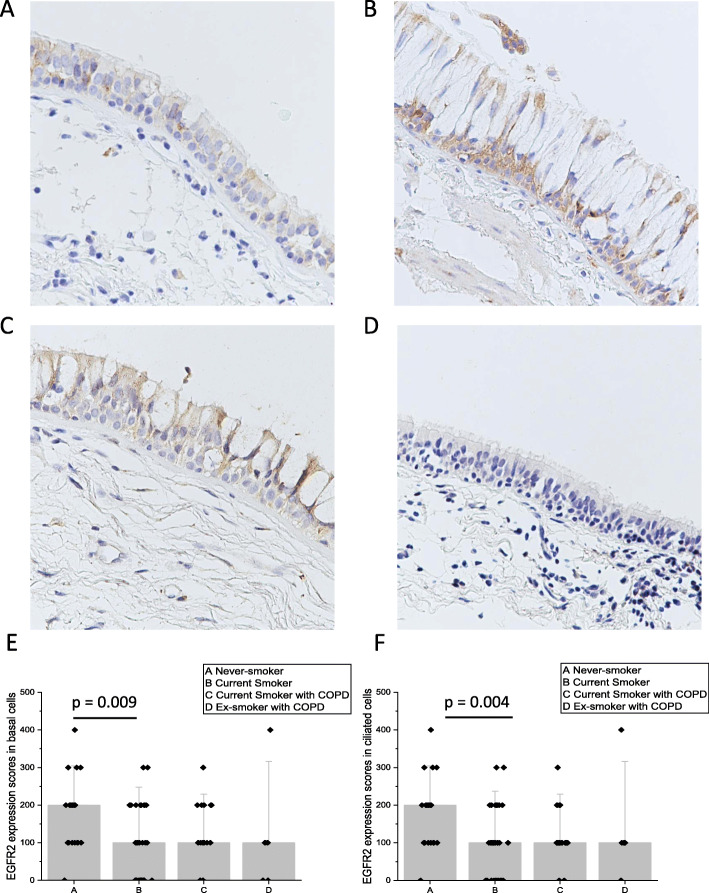
Representative images of the immunohistochemical stainings for EGFR 2. Expression of EGFR2 in cells suggesting basal cell and ciliated cell phenotype in the large airways of a Never-smoker with normal lung functions (**a**), a Smoker with normal lung function (**b**), MUC1 expression in basal cells of an ex-smoker with COPD (**c**) and a Smoker with COPD (**d**). EGFR2 scores of all subjects in basal cell phenotype (**e**) and in ciliated cell phenotype (**f**). Results are shown as median bars and standard error of median

EGFR2 was detected in the cytosol of both the cells suggesting basal and ciliated cell phenotype of the airway epithelium (Fig. 6a-d). Sub-mucosal glands, if present, were also positive for EGFR2. EGFR2 expression was higher in the Never-smokers as compared to the Smoker group both in the basal phenotype (*p* = 0.009) and ciliated cell phenotype (*p* = 0.004) (Fig. 6e-f).

### Soluble MUC1 in bronchial wash samples in smoking and ex-smoking COPD

**Fig. 7:**
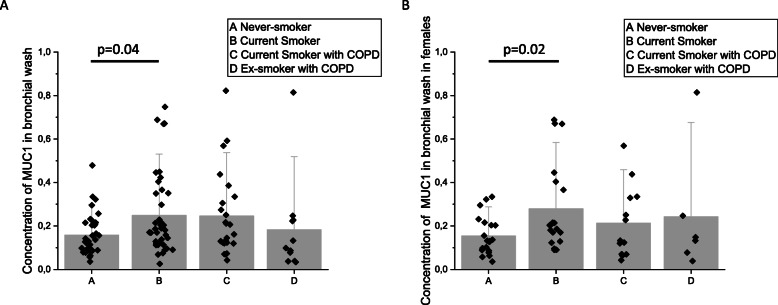
Soluble MUC1 from basal cells or sub-epithelial glands were measured with quantitative immunoblotting. MUC1 concentration in bronchial wash samples. MUC1 concentration in the whole group (**a**) and in the females (**b**). 1 unit equals 0.4 μg/mL. Results are shown as mean and standard error of mean

The concentration of soluble MUC1 was analysed with immunoblotting of bronchial wash samples. Smokers and Smokers with COPD had a higher concentration of soluble MUC1 in their bronchial wash samples than Never-smokers and ex-smokers with COPD, respectively. A statistically significant difference was detected between Never-smokers and Smokers both in the joint gender analysis (Fig. 7a; *p* = 0.04), and in females (Fig. 7b; *p* = 0.02), but not in males although the tendency is similar to females (*p* = 0.2).

### Effect of smoking on the expression of MUC1, MUC4, EGFR1 and EGFR2

**Fig. 8:**
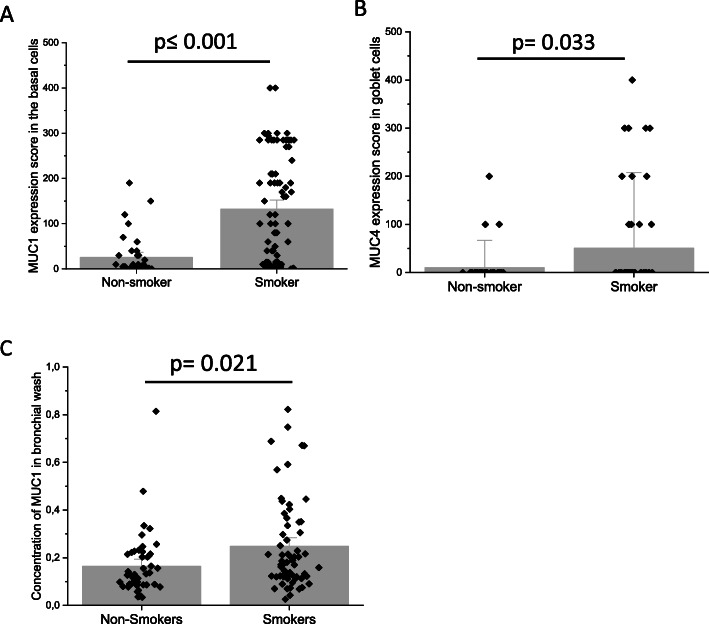
Cell-bound and soluble MUC1 as well as MUC4 was studied in large airways of non-smokers and smokers. The non-smoker group includes both never-smokers and COPD ex-smokers whereas the smoker group includes smokers with normal lung function and smokers with COPD. **a** MUC1 scores in cells suggesting basal cell phenotype from immunohistochemical stainings; **b** MUC4 scores in cells suggesting goblet cell phenotype from immunohistochemical stainings and **c**. Amount of MUC1 in bronchial wash samples; 1 unit equals 0.4 μg/mL. MUC1 results are shown as mean bars and standard error of mean, MUC4 results are shown as median bars and standard error of median

The expression level of cell-bound MUC1 from bronchial mucosal biopsies (Fig. 8a) was higher in smokers (with and without COPD) than non-smokers (including both never-smoker controls and ex-smoker COPD patients) (*p* ≤ 0.001). Cell-bound MUC4 expression (Fig. 8b) was higher in smokers as compared to non-smokers in goblet cells (*p* = 0.03). A higher concentration of soluble MUC1 (Fig. 8c) was found in bronchial wash samples from the large airways of smokers when compared to non-smokers (*p* = 0.0021). Interestingly, the amount of EGFR2 expression was higher in non-smokers as compared to smokers in basal cells (*p* = 0.031) and in ciliated cells (*p* = 0.020).

### MUC1 expression in patients with Chronic Bronchitis

**Fig. 9:**
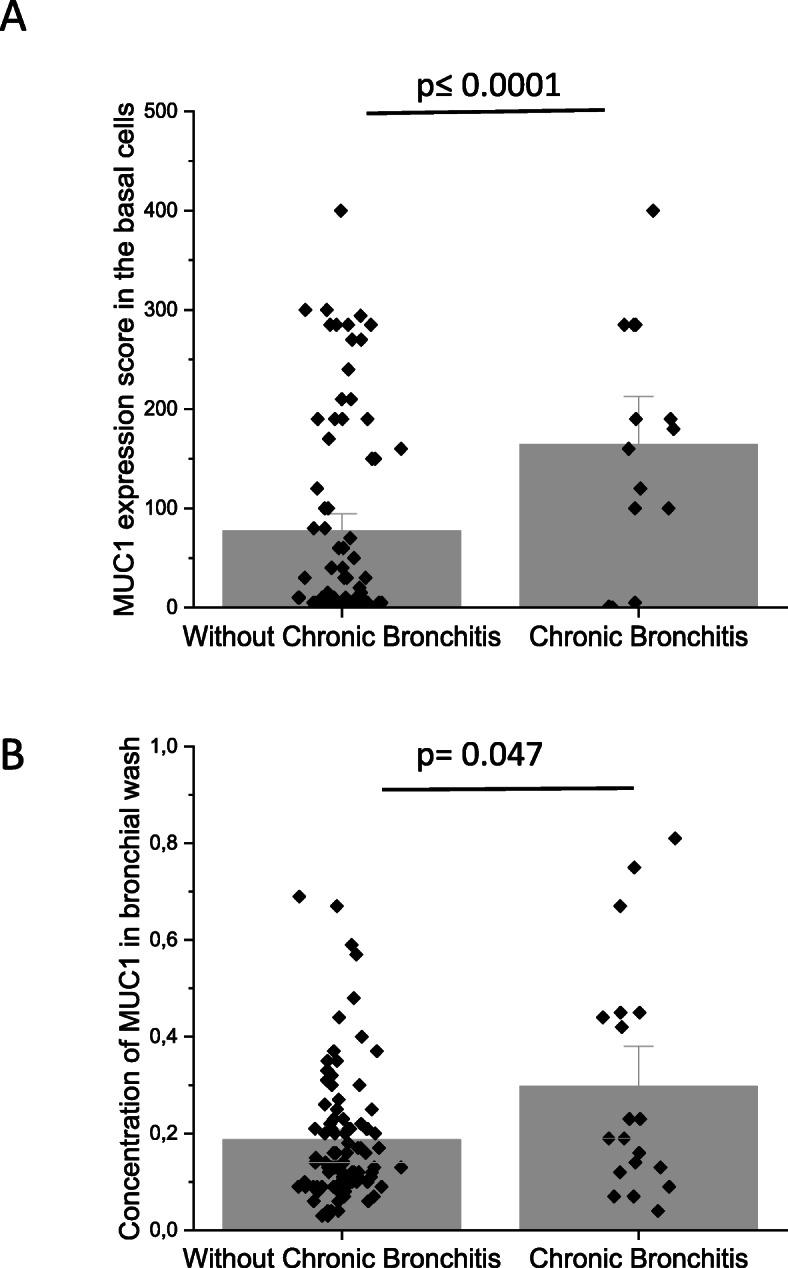
Differences in MUC1 expression levels in patients with or without chronic bronchitis. **a** Scores of cell-bound MUC1 expression in large airways and **b**. Amount of MUC1 in bronchial wash samples from the patients; 1 unit equals 0.4 μg/mL. MUC1 results are shown as mean bars and standard error, MUC1 results are shown as median bars and standard error of median

The expression of cell-bound and the concentration of soluble MUC1 were higher in subjects with chronic bronchitis as compared to non-bronchitic patients (Fig. 9, *p* ≤ 0.0001 and *p* = 0.047, respectively). The level of MUC1 expression in large airways was higher in females with chronic bronchitis (*p* = 0.033) and these women had a higher level of soluble MUC1 (*p* = 0.021), but these phenomena were not evident in males (*p* = 0.21). The diagnosis of chronic bronchitis was not associated with MUC4, EGFR1 or EGFR2 expression.

### MUC1 expression associated with lung function

The level of cell-bound MUC1 expression negatively correlated with post-bronchodilator measures of lung functions when all cases were pooled together. The values of FEV_1_% of predicted, FEV_1_/VC and the FEV_1_/FVC ratios negatively correlated with the level of MUC1 expression (*R* = -0.24, *p* = 0.018, *R* = -0.23, *p* = 0.027 and R = -0.29, *p* = 0.004, respectively). A negative correlation was observed also between MUC1 expression and lung functions (FEV_1_% (*R* = -0.35, *p* = 0.015) and FEV_1_/FVC ratio (*R* = -0.36, *p* = 0.013)) in all males pooled together, but not in all females pooled together.

### MUC1 expression associated with smoking

The extent of MUC1 expression correlated both with smoking history measured as pack-years (*R* = 0.48, *p* ≤ 0.001) and with current cigarette consumption (*R* = 0.40, *p* ≤ 0.001). There was a positive correlation observed between pack-years and MUC1 expression in both the male and female groups (*R* = 0.62, *p* ≤ 0.001 and *R* = 0.31, *p* = 0.04, respectively). Current cigarette consumption was positively correlated with MUC1 expression in the male group (*R* = 0.53, *p* ≤ 0.001), but not in the females.

### MUC4 expression associated with smoking

Current cigarette consumption was positively correlated with MUC4 expression in cells suggesting goblet cell phenotype (*R* = 0.23, *p* = 0.031). This correlation was primarily attributable to the males (*R* = 0.34, *p* = 0.02), and not to the females (*p* = 0.31).

### EGFR2 correlated with positive expression of MUC4 in goblet cells

EGFR2 expression in the cells with basal and ciliated cell phenotype negatively correlated with the level of MUC4 expression in the cells suggesting goblet cell phenotype (*R* = − 0.29, *p* = 0.01 and *R* = -0.29, *p* = 0.01, respectively). This was primarily driven by the females (*R* = -0.32, *p* = 0.04 and *R* = -0.32, *p* = 0.04), with no significant associations evident in males. EGFR1 levels did not correlate with MUC1 or MUC4. EGFR2 levels did not correlate with MUC1 expression.

### EGFR2, but not EGFR1, correlated with smoking and lung functions

EGFR2 expression in the cells with basal cell and ciliated cell phenotype correlated negatively with the number of pack-years smoked (*R* = -0.26, *p* = 0.02 and *R* = − 0.27, *p* = 0.01, respectively), and with current cigarette consumption (*R* = -0.24, *p* = 0.03 and *R* = -0.24, *p* = 0.03, respectively) in all Smokers. In the male group, the EGFR2 expression correlated negatively with the current consumption of cigarettes (*R* = -0.38, *p* = 0.01, for both cell phenotypes), but not with the parameters reflecting lung functions or with smoking.

A compilation of the cell-specific immunohistochemical results and demographic associations is shown in Table 3.

**Table 3:** Compilation of the cell-specific immunohistochemical results and demographic associations

Factor	Airway cell phenotype	Demographic association
MUC1	Basal cell	↑ Smokers and Chronic bronchitis Correlated negatively with lung functions in male Smokers Correlated positively with pack-years
MUC4	Goblet cells	↑ Smokers
EGFR1	Basal cells	No change
EGFR2	Basal and ciliated cells	↑ Healthy Correlated positively with lung functions Correlated negatively with pack-years and current cigarette consumption

Alterations in abundance are expressed in comparison to the healthy Never-smoker group

## Discussion

We investigated both cell-specific immunohistochemical expression and the concentration of soluble MUC1 in human airways from age- and gender-matched healthy never-smokers (Never-smoker), current smokers with normal lung function (Smoker) and mild-moderate COPD-patients. We observed that smoking and a diagnosis of chronic bronchitis, but not airway obstruction, were associated both with increased MUC1 expression in cells suggesting basal cell phenotype, as well as the amount of soluble MUC1 protein in the airway lumen. The sources of the soluble MUC1 are likely to be secretions from the sub-epithelial glands, shedding of membrane bound MUC1 from epithelial cells or cells of hematopoietic origin [13, 38, 39]. Previous investigations of MUCs in COPD have focused mostly on MUC5A and MUC5B, whereas there are far fewer investigations of the membrane-bound MUCs such as MUC1 and MUC4. These present results and other studies have shown that the cellular localization of various types of MUCs in airways is variable whereas some MUC types, such as MUC1, seem rather to specifically originate from a certain cell type, namely basal cells.

Caramori et al. investigated MUC5A and 5B in bronchial rings of large airways from patients that had undergone surgery for lung cancer and detected different expressions of MUC5AC and MUC5B in central airways [40]. Kovalenko et al. utilized bronchial biopsies for the assessment of MUC2, MUC3 and MUC4 in large airways during an acute exacerbation of COPD, revealing that the expressions of both MUC2 and MUC3 were reduced [41]. O’Donnell et al. found that the levels of EGFR, ErB3 and MUC5AC but not MUC2 and MUC5B, were increased in bronchial biopsies of smokers as compared to non-smokers and COPD patients (*n* = 51) [15]. We found that MUC1 was localized in the cells suggesting basal cell phenotype of large airways whereas all the other epithelial cell types were negative. This was both an unexpected and a novel finding since the cell-specific localization of MUC1 protein in the large airways has not been previously published. The basal cell-origin expression for MUC1 was something of a surprise since this cell type is believed to act as a progenitor cell in the airways. The amount of KL-6, a mucinous sialylated sugar chain on the extracellular domain of human MUC1 protein, has been also found to be higher both in serum and in sputum of stable COPD patients [42] as well as during COPD exacerbations [43]. In peripheral lung, KL-6 is predominantly expressed in type II alveolar cells [44] and it has been shown to be highly expressed in bronchoalveolar lavage (BAL) fluid samples obtained from sarcoidosis patients [45]. Serum levels of KL-6 are known to be elevated in a variety of interstitial lung diseases including idiopathic pulmonary fibrosis; these diseases are characterized by alveolar epithelial cell damage [46]. An increased KL-6 level in lung carcinoma patients has been associated with a poor prognosis [47] and downregulation of the MUC1 was claimed to inhibit the non-small cell lung carcinoma progression [48]. In summary, it can be speculated that MUC1 may be a marker of lung progenitor cells both in airways and in alveoli, as MUC1 is predominantly expressed in the two cell types assumed to exhibit the properties of lung stem cells, namely basal cells of airways and type II alveolar cells [49].

Caramori et al. observed that MUC4 was expressed in all cell types present in the airways [50]. Our results partially confirm this finding, i.e. basal and ciliated cells were positive for MUC4, but in contrast, in that publication cells suggesting goblet cell phenotype were negative in most of the cases. Here, immunohistochemical reactivity for MUC4 in goblet cells was evident. The level of MUC4 expression was positively associated with smoking history, but not with lung function. In contrast to the Caramori study, Kovalenko et al. were unable to detect any positivity for MUC4 in the airway cells of COPD patients with GOLD stage III [41]. Similar to MUC1, most of the previous studies on MUC4 have been directed at lung cancer whereas investigations into airway disorders are sparse.

Our results revealed that soluble MUC1 protein levels were higher in the bronchial secretions of Smokers than in Never-smokers. Interestingly, Ishikawa et al. [42] identified higher levels of KL-6 both in sputum and plasma in smokers as compared to non-smokers, these results are in line with our findings of elevated levels of MUC1 in bronchial wash [42].

The level of MUC1 expression correlated with lung function and was increased in individuals with chronic bronchitis, especially in females. Chronic bronchitis has been suggested to be more common among females than males [51].

Previous studies have revealed that the suppression of MUC1 led to a down-regulation of the expression of EGFR, and moreover, MUC1 regulated the nuclear location and function of EGFR [52]. Exposure to cigarette smoke up-regulated EGFR mRNA expression and induced the activation of EGFR-specific tyrosine phosphorylation, resulting in an up-regulation of MUC5AC mRNA and protein production in an in vitro-model; these effects were inhibited by exposure to selective EGFR tyrosine kinase inhibitors [15]. Anagnostis et al. demonstrated that EGRF1, 2 and 3 mRNA levels were higher in COPD patients as compared to healthy smokers [53]. The largest immunohistochemical study so far performed showed that the level of EGFR was increased in goblet and ciliated cells in large airways of smokers irrespective of the presence of COPD [15]. Our results are at odds with the previous findings. In our subjects, EGFR2 protein expression was actually decreased in the smokers as compared to the never-smokers and the decrease correlated with smoking habits, whereas EGFR1 expression did not reveal any demographic correlations. Similar to the present work, a recent study investigated both EGFR1 and EGFR2 with immunohistochemistry, showing higher EGFR1 expression in ex-smokers with COPD than in ex-smokers without COPD [54]. It has been reported that HER2, a.k.a. EGFR2 was more frequent in COPD patients in comparison with lung cancer sufferers [55]. One clinical trial examining inhaled EGFR reported negative results since a four-week treatment did not decrease the epithelial levels of MUC2, MUC5A and MUC5B in the bronchial biopsies [56]. A recent study using microarray analysis showed that the numbers of EGF receptors were increased in COPD as compared to smokers without COPD [53]. Interestingly, our results indicated that the level of MUC4 expression in goblet cells correlated with that of EGFR2 while the expression of EGFR1 did not correlate with that of MUC1, which is in line with a previous report where the level of MUC4 expression correlated with that of EGFR2 in lung carcinoma specimens [57].

It has been suggested that the phenotype of the disease differs between genders, and we have previously reported evidence of molecular-level sex differences in the Karolinska COSMIC cohort i.e. in the levels of the proteome [25, 58], eicosanoid [21], metabolome [23] and cytokine concentrations [22]. Based on reports of an involvement of the estrogen receptor in the regulation of MUC1 expression [59], we also performed sex-stratified analyses. We found that smoking increased MUC1 expression in the large airways to a greater extent in men than in women, and this correlated negatively with lung function only in males although the tendency was the same in female group. In female smokers, the concentration of soluble MUC1 in the airway lumen was higher as compared to the healthy never-smoker group. Female subjects with chronic bronchitis had higher levels of MUC1 and MUC4 in comparison with the subjects without chronic bronchitis. Interestingly same tendency was seen in males but the differences did not statistically significant.

There are some limitations to this study. The group sizes in general are relatively small in particular in the COPD group, where the numbers of current smokers are not balanced with ex-smokers. Also, a major limitation is that the study is based only on biopsies from the large airways. In spite of these concerns, the results are significant, and the gender differences are supported by other data blocks from the same cohort [21–30].

In conclusion, we detected an association of MUC1 and MUC4 expression with smoking and with chronic bronchitis, but not with airway obstruction. Our results support the hypothesis that mucins are important in development of airway symptoms due to smoking. Further studies to elucidate the contributions of each MUC-type in the pathogenesis of COPD would be beneficial.

## Supplementary information

**Additional file 1: Figure S1.** Immunohistochemical stainings performed with consecutive tissue. Sections were cut in consecutive order, and stained in carefully selected order. In Images A-B p63 and MUC1 staining from healthy current smoker is presented. Basal cells were detected with a specific antibody, p63, which stains nucleus in basal cells showing a dark brown staining (A). Positive MUC1 staining in the basal cells with the score of 285 (B). Cytosolic positivity of the basal cells was considered significant. Red arrows show the staining in the basal cells. In Images C-D AB-PAS and MUC4 stainings from healthy current smoker is presented. Goblet cells were detected with AB-PAS staining, in which goblet cells are stained in clear blue (C). Positive MUC4 staining in all cell types with scores of 300 (D). All the airway epithelial cells types are MUC4 positive, as well as the endothelial cells in small veins. Cytosolic positivity was considered significant. Thin black arrows in both C and D images show the goblet cells and green arrows point to the small veins. **Table S1.** Comparison between the groups. Scoring of the immunohistochemical stainings mucins and EGFRs with *p*-value and FDR value. *p* ≤ 0.05 and FDR ≤0.2 were considered significant. **Table S2.** Comparison between the groups. Concentration of the soluble MUC1 in bronchial wash with p-value and FDR value. p ≤ 0.05 and FDR ≤0.2 were considered significant. **Table S3.** Results of correlations between mucins, EGFRs, lung functions and smoking history. p ≤ 0.05 and FDR ≤0.2 were considered significant.

## Data Availability

The datasets generated and/or analyzed in the current study are not publicly available due to state restrictions. Thus, information may compromise research participant privacy/consent but data are available from the corresponding author on reasonable request.

## Abbreviations

AB-PAS: Alcian Blue periodic acid-Schiff
BAL: Bronchoalveolar lavage
COPD: Chronic obstructive pulmonary disease
DAB: Diaminobenzidine
GOLD: The Global Initiative for Chronic Obstructive Lung Disease
EGFR: Epithelial growth factor receptor
EMA: Epithelial membrane antigen
FEV1: Forced expiratory volume
FVC: Forced vital capacity
HE: Haematoxylin-eosin
KL-6: Krebs von den Lungen antigen
mRNA: messenger ribonucleic acid
MUC: Mucin
PBS: Phosphate-buffered saline
VC: Vital capacity
