# Supplementary material for: Smoking-associated increase in mucins 1 and 4 in human airways
Source: Respir Res. 2020 Sep 18;21:239. doi: 10.1186/s12931-020-01498-7 (PMC7499856; doi:10.1186/s12931-020-01498-7)
Supplement: Supplementary file 1 — Additional file 1: Figure S1. Immunohistochemical stainings performed with consecutive tissue. Sections were cut in consecutive order, and stained in carefully selected order. In Images A-B p63 and MUC1 staining from healthy current smoker is presented. Basal cells were detected with a specific antibody, p63, which stains nucleus in basal cells showing a dark brown staining (A). Positive MUC1 staining in the basal cells with the score of 285 (B). Cytosolic positivity of the basal cells was considered significant. Red arrows show the staining in the basal cells. In Images C-D AB-PAS and MUC4 stainings from healthy current smoker is presented. Goblet cells were detected with AB-PAS staining, in which goblet cells are stained in clear blue (C). Positive MUC4 staining in all cell types with scores of 300 (D). All the airway epithelial cells types are MUC4 positive, as well as the endothelial cells in small veins. Cytosolic positivity was considered significant. Thin black arrows in both C and D images show the goblet cells and green arrows point to the small veins. Table S1. Comparison between the groups. Scoring of the immunohistochemical stainings mucins and EGFRs with p-value and FDR value. p ≤ 0.05 and FDR ≤0.2 were considered significant. Table S2. Comparison between the groups. Concentration of the soluble MUC1 in bronchial wash with p-value and FDR value. p ≤ 0.05 and FDR ≤0.2 were considered significant. Table S3. Results of correlations between mucins, EGFRs, lung functions and smoking history. p ≤ 0.05 and FDR ≤0.2 were considered significant. [file 12931_2020_1498_MOESM1_ESM.zip › Correction/supplementary files/Supplemental_data_ESM.docx]

Supplemental Data

Figure Legend

Figure 1. Immunohistochemical stainings performed with consecutive tissue. Sections were cut in consecutive order, and stained in carefully selected order. In Images A-B p63 and MUC1 staining from healthy current smoker is presented. Basal cells were detected with a specific antibody, p63, which stains nucleus in basal cells showing a dark brown staining (A). Positive MUC1 staining in the basal cells with the score of 285 (B). Cytosolic positivity of the basal cells was considered significant. Red arrows show the staining in the basal cells. In Images C-D AB-PAS and MUC4 stainings from healthy current smoker is presented. Goblet cells were detected with AB-PAS staining, in which goblet cells are stained in clear blue (C). Positive MUC4 staining in all cell types with scores of 300 (D). All the airway epithelial cells types are MUC4 positive, as well as the endothelial cells in small veins. Cytosolic positivity was considered significant. Thin black arrows in both C and D images show the goblet cells and green arrows point to the small veins.

Table 1. Comparison between the groups. Scoring of the immunohistochemical stainings mucins and EGFRs with p-value and FDR value. p ≤0.05 and FDR ≤0.2 were considered significant.

|  | Non-smoker vs smokers | | Comparison with and without chronic bronchitis | | Never-smoker vs. Current Smokers | | Current smokers vs. Current smokers with COPD | | Smokers with COPD vs. Ex-smokers with COPD | |
| --- | --- | --- | --- | --- | --- | --- | --- | --- | --- | --- |
| All | p | FDR | p | FDR | p | FDR | p | FDR | p | FDR |
| MUC1 Score | 0,000 | 0,000 | 0,000 | 0,001 | 0,000 | 0,003 | 0,297 | 0,557 | 0,027 | 0,149 |
| MUC4 Score in Basal cells | 0,057 | 0,230 | 0,219 | 0,486 | 0,047 | 0,207 | 0,406 | 0,634 | 0,528 | 0,718 |
| MUC4 Score in Goblet cells | 0,033 | 0,168 | 0,075 | 0,266 | 0,009 | 0,061 | 0,468 | 0,670 | 0,845 | 0,895 |
| MUC4 score in Ciliated cells | 0,244 | 0,509 | 0,620 | 0,787 | 0,196 | 0,452 | 0,507 | 0,700 | 0,769 | 0,857 |
| EGFR Score | 0,589 | 0,771 | 0,302 | 0,564 | 0,715 | 0,838 | 0,080 | 0,272 | 0,242 | 0,509 |
| EGFR2 Score in Basal cells | 0,031 | 0,162 | 0,028 | 0,153 | 0,009 | 0,061 | 0,345 | 0,590 | 0,318 | 0,573 |
| EGFR2 Score in Ciliated cells | 0,020 | 0,114 | 0,015 | 0,090 | 0,004 | 0,034 | 0,246 | 0,510 | 0,318 | 0,573 |
|  |  |  |  |  |  |  |  |  |  |  |
| Males |  |  |  |  |  |  |  |  |  |  |
| MUC1 Score | 0,000 | 0,001 | 0,001 | 0,010 | 0,001 | 0,011 | 0,625 | 0,788 | 0,071 | 0,258 |
| MUC4 Score in Basal cells | 0,169 | 0,424 | 0,457 | 0,664 | 0,200 | 0,458 | 0,706 | 0,838 | 0,470 | 0,670 |
| MUC4 Score in Goblet cells | 0,064 | 0,241 | 0,141 | 0,374 | 0,043 | 0,200 | 0,255 | 0,513 | 0,564 | 0,750 |
| MUC4 score in Ciliated cells | 0,141 | 0,374 | 0,203 | 0,462 | 0,489 | 0,691 | 0,844 | 0,895 | 0,031 | 0,162 |
| EGFR Score | 0,314 | 0,573 | 0,440 | 0,656 | 0,647 | 0,805 | 0,413 | 0,636 | 0,837 | 0,895 |
| EGFR2 Score in Basal cells | 0,008 | 0,059 | 0,038 | 0,187 | 0,010 | 0,068 | 0,170 | 0,424 | 0,356 | 0,593 |
| EGFR2 Score in Ciliated cells | 0,008 | 0,059 | 0,038 | 0,187 | 0,010 | 0,068 | 0,170 | 0,424 | 0,356 | 0,593 |
|  |  |  |  |  |  |  |  |  |  |  |
| Female |  |  |  |  |  |  |  |  |  |  |
| MUC1 Score | 0,007 | 0,059 | 0,033 | 0,168 | 0,014 | 0,088 | 0,407 | 0,634 | 0,294 | 0,553 |
| MUC4 Score in Basal cells | 0,177 | 0,427 | 0,501 | 0,695 | 0,139 | 0,374 | 0,468 | 0,670 | 0,774 | 0,860 |
| MUC4 Score in Goblet cells | 0,237 | 0,509 | 0,238 | 0,509 | 0,072 | 0,260 | 0,913 | 0,943 | 0,808 | 0,884 |
| MUC4 score in Ciliated cells | 0,851 | 0,897 | 0,428 | 0,645 | 0,251 | 0,511 | 0,241 | 0,509 | 0,262 | 0,519 |
| EGFR Score | 0,767 | 0,857 | 0,597 | 0,775 | 0,419 | 0,637 | 0,196 | 0,452 | 0,344 | 0,590 |
| EGFR2 Score in Basal cells | 0,602 | 0,776 | 0,181 | 0,431 | 0,249 | 0,511 | 0,851 | 0,897 | 0,138 | 0,374 |
| EGFR2 Score in Ciliated cells | 0,454 | 0,664 | 0,122 | 0,350 | 0,130 | 0,362 | 0,702 | 0,838 | 0,138 | 0,374 |

Table 2.Comparison between the groups. Concentration of the soluble MUC1 in bronchial wash with p-value and FDR value. p ≤0.05 and FDR ≤0.2 were considered significant.

|  | Non-smoker vs smokers | | Comparison with and without chronic bronchitis | | Never-smoker vs. Current Smokers | |
| --- | --- | --- | --- | --- | --- | --- |
|  | p | FDR | p | FDR | p | FDR |
| All | 0,021 | 0,121 | 0,047 | 0,204 | 0,044 | 0,203 |
| Male | 0,230 | 0,499 | 0,410 | 0,629 | 0,200 | 0,453 |
| Female | 0,260 | 0,511 | 0,021 | 0,13 | 0,020 | 0,117 |

Table 3. Results of correlations between mucins, EGFRs, lung functions and smoking history. p ≤0.05 and FDR ≤0.2 were considered significant.

| Correlations between | Values | All | Male | Female |
| --- | --- | --- | --- | --- |
| MUC4 Score and MUC1 Score | R | 0,34 | 0,36 | 0,34 |
|  | p | 0,03 | 0,01 | 0,03 |
|  | FDR | 0,034 | 0,24 | 0,4 |
| FEV1% and MUC1 Score | R | -0,24 | -0,35 | -0,12 |
|  | p | 0,018 | 0,015 | 0,38 |
|  | FDR | 0,2 | 0,24 | 0,88 |
| FEV1/VC and MUC1 Score | R | -0,23 | -0,29 | -0,15 |
|  | p | 0,027 | 0,04 | 0,28 |
|  | FDR | 0,25 | 0,41 | 0,82 |
| FEV1/FVC and MUC1 Score | R | -0,29 | -0,36 | -0,24 |
|  | p | 0,004 | 0,013 | 0,09 |
|  | FDR | 0,08 | 0,22 | 0,63 |
| Packyears and MUC1 Score | R | 0,48 | 0,62 | 0,31 |
|  | p | ≤0.001 | ≤0.001 | 0,04 |
|  | FDR | ≤0.001 | ≤0.001 | 0,2 |
| Cigarette consumption and MUC1 Score | R | 0,4 | 0,53 | 0,24 |
|  | p | ≤0.001 | ≤0.001 | 0,1 |
|  | FDR | 0,002 | 0,01 | 0,63 |
| Cigarette consumption and MUC4 Score in goblet cells | R | 0,23 | 0,34 | 0,15 |
|  | p | 0,031 | 0,02 | 0,31 |
|  | FDR | 0,26 | 0,28 | 0,83 |
| EGFR2 in basal cells and MUC4 in goblet cells | R | -0,29 | -0,22 | -0,32 |
|  | p | 0,01 | 0,18 | 0,04 |
|  | FDR | 0,17 | 0,64 | 0,46 |
| EGFR2 in ciliated cells and MUC4 in goblet cells | R | -0,29 | -0,22 | -0,32 |
|  | p | 0,01 | 0,18 | 0,04 |
|  | FDR | 0,17 | 0,64 | 0,46 |
| Packyears and EGFR2 in basal cells | R | -0,26 | 0,26 | -0,25 |
|  | p | 0,02 | 0,08 | 0,12 |
|  | FDR | 0,19 | 0,48 | 0,63 |
| Packyears and EGFR2 in ciliated cells | R | -0,27 | -0,26 | -0,28 |
|  | p | 0,01 | 0,08 | 0,06 |
|  | FDR | 0,16 | 0,48 | 0,55 |
| Cigarette consumption and EGFR2 in basal cells | R | -0,24 | -0,38 | -0,09 |
|  | p | 0,03 | 0,01 | 0,53 |
|  | FDR | 0,25 | 0,22 | 0,92 |
| Cigarette consumption and EGFR2 in ciliated cells | R | -0,24 | -0,38 | -0,09 |
|  | p | 0,03 | 0,01 | 0,53 |
|  | FDR | 0,25 | 0,22 | 0,92 |
